# Public, environmental, and occupational health research activity in Arab countries: bibliometric, citation, and collaboration analysis

**DOI:** 10.1186/2049-3258-73-1

**Published:** 2015-01-05

**Authors:** Waleed M Sweileh, Sa’ed H Zyoud, Samah W Al-Jabi, Ansam F Sawalha

**Affiliations:** 1 Department of Pharmacology and Toxicology, College of Medicine and Health Sciences, An-Najah National University, Nablus, Palestine; 2 Department of Clinical and Community Pharmacy, College of Medicine and Health Sciences, An-Najah National University, Nablus, Palestine

**Keywords:** Arab countries, Public health, Environmental and occupational health, Bibliometric analysis, ISI web of science

## Abstract

**Background:**

The objective of this study was to analyze quantity, assess quality, and investigate international collaboration in research from Arab countries in the field of public, environmental and occupational health.

**Methods:**

Original scientific articles and reviews published from the 22 Arab countries in the category "public, environmental & occupational health" during the study period (1900 – 2012) were screened using the ISI Web of Science database.

**Results:**

The total number of original and review research articles published in the category of "public, environmental & occupational health" from Arab countries was 4673. Main area of research was tropical medicine (1862; 39.85%). Egypt with 1200 documents (25.86%) ranked first in quantity and ranked first in quality of publications (*h*-index = 51). The study identified 2036 (43.57%) documents with international collaboration. Arab countries actively collaborated with authors in Western Europe (22.91%) and North America (21.04%). Most of the documents (79.9%) were published in public health related journals while 21% of the documents were published in journals pertaining to prevention medicine, environmental, occupational health and epidemiology.

**Conclusion:**

Research in public, environmental and occupational health in Arab countries is in the rise. Public health research was dominant while environmental and occupation health research was relatively low. International collaboration was a good tool for increasing research quantity and quality.

## Background

Public, environmental and occupational health is an important medical discipline. Studies and research in this field can shed light on the current health situation, future health challenges, epidemiology of diseases, health policies, and standards of healthcare in any particular country. Therefore, assessment of the research activity in this field is important for several reasons. First, it enables healthy policy makers to aware of the national health status, health indicators, and health challenges facing the country [1, 2]. Second, it reflects the contribution of the country to global scientific literature [3–9]. Third, it reflects the extent of the country’s collaboration with international investigators in the field of health research which is an important tool in improving national research activity and quality [10, 11]. Some authors described the field of public health as multidisciplinary, high-benefit, but undervalued and that researchers and practitioners need to influence the development of public health research within individual countries to promote public health research area [12].

Many health issues in Arab countries are considered serious and research and collaboration in these health issues is a key factor in facing and solving these health issues. For example, North Africa, Middle East, and Gulf area will have the second highest increase in percentage of people with diabetes mellitus in 2030 compared to other parts of the world [13]. According to International Diabetes Federation (IDF), 6 out of the world’s top ten countries for highest prevalence (%) of diabetes are in the Middle East and North Africa Region – Kuwait, Lebanon, Qatar, Saudi Arabia, Bahrain and the United Arab Emirates [14]. A recently published review article indicated that the overall estimated prevalence of hypertension among Arabs was 29.5% which is higher than that reported from the USA and sub-Saharan African [15]. Another recent study indicated that prevalence of hypertension among Arab American is 36.5% which is higher than the overall worldwide estimates of hypertension prevalence [16]. Furthermore, reports from some Arab countries indicated that hypertension is being under-treated and mostly uncontrolled [17–23]. Several studies reported that the prevalence rate of obesity in Arabic region is high [24–26]. Arab countries have been the source of some fatal infectious diseases such as Middle East Respiratory Syndrome which was initially diagnosed in Kingdom of Saudi Arabia (KSA). There was an outbreak of polio in Syria that made international agencies to call for ceasefire to accomplish polio vaccination campaign [27]. Another example is the cholera outbreak in Baghdad after the second gulf war [28]. According to World Health Organization (WHO), conservative communities like Arab countries have the fastest rate of increase in the number of HIV infections and the lowest coverage with antiretroviral therapy [29]. Highly pathogenic and serious viral infections like avian flu virus, hepatitis B and hepatitis C are still an important risk of morbidity and mortality and pose a real threat for some Arab countries like Egypt [30–34]. Similarly, serious parasitic infections like malaria, schistosomiasis and trypanosomiasis constitute major health, social and economic challenge for Egypt, Sudan, Yemen and other Arab countries [35–40]. Zoontic infections, like brucellosis and hydatid disease, are also present in several Arab countries and pose a continuous health challenge [41–45]. In addition to all of this, there is evidence that serious and common infectious agents in Arab region, like Mycobacterium tuberculosis and Staphylococcus aureus and some gram-negative bacilli are developing multiple drug resistance which is a true future public health challenge at regional and global level [46–54].

Several studies about public health situation, challenges and future aspects of health systems in Arab countries have been published [55–57]. Similarly, several studies in environmental and occupational health risks in Arab countries have also been published [58–63]. However, up to the authors’ best knowledge, no reports about the quantity and quality of public, environmental and occupational health from Arab countries had been published [64–67]. Bibliometric studies are important because they can be used to obtain data and draw a picture about the state of research in any particular field in any particular country. Several international bibliometric studies in the field of public, environmental and occupational health have been published but none was published from Arab countries [67–69]. Therefore, this study was conducted to analyze the quantity and quality of research published in "Public, Environmental & Occupational Health" category in Arab countries. These disciplines are joined in one category in Web of Science (WoS) database which we used in data extraction methodology. This category encompasses all journals in the fields of public health, occupational health, environmental health, prevention medicine, and epidemiology. Few years ago, this category was called "Preventive and Occupational Medicine, Epidemiology and Public Health". Several bibliometric analysis were published about various medical fields from Arab countries but none was published about public, environmental and occupational health [3, 70, 71]. Our study comes in line with several recent articles published in Lancet journal that described the health situation in Arab countries and strongly encouraged researchers to publish in this field [29, 72]. Therefore, such study will shed light on the current and future "Public, Environmental, and Occupational Health" status in Arab countries. Furthermore, the results of the study will help public health advocates, environmentalists, toxicologists and people in politics as well to decide on future research in this field and how to allocate funds for researchers in this field. In addition, the momentum for research activity needs to be maintained through continuous analysis of publications from researchers in the region to provide feedback to academic, health institutions and education planners. Finally, in Arab countries, health priorities are changing [73–78] which necessitates periodic assessments of health challenges and progress in health research especially when political and humanitarian challenges are dominating the scene in many Arab countries [79–83].

## Methods

The data used in this study were based on the ISI Web of Science, which is one of the world largest databases of peer-reviewed literature. The WoS databases provide authoritative, multidisciplinary coverage from more than 12,000 high impact research journals worldwide [84]. Web of Science was developed by Thomson Scientific and is well known for its annual report about journal impact factor which is an important measure of the quality and influential power of publications. Web of Science is easy to use and has a simple and advanced search tools. With the advanced search tool, a list of codes can be used to achieve the required objective of the search. One of the codes in WoS advanced search is "WC" which refers to Web of Science Category. There are more than 100 categories for selection and one of which is "public, environmental, and occupational health". Few years ago, this category was called "Preventive and Occupational Medicine, Epidemiology and Public Health" because it encompasses journals in all fields listed in the category. In addition to the above character of the advanced search tool in WoS, detailed citation analysis of the results of the advanced search can also be easily obtained. A comprehensive analysis of the advantages and disadvantages of various databases including Web of Science, PubMed, and Scopus is presented by Falagas et al. [85]. In contrast to PubMed, Web of Science covers most scientific publication and not only the medical and biomedical publication as in the case of PubMed. Furthermore, Web of Science covers the oldest publications and its records go back to 1900. A major disadvantage of PubMed is the fact that it does not provide citation analysis and therefore does not allow for qualitative analysis of published literature [85]. In the current study, the objective of the study was to assess the quantity and quality of public, environmental and occupational health related research from Arab countries. One of our objectives is qualitative analysis which cannot be carried out by PubMed. Furthermore, although public/environmental/ occupational health is a medical subject, research in this field could have been carried out and published in non-medical journals which make PubMed less suitable for this purpose than WoS. Finally, WoS has journal categories that would facilitate the accomplishment of the objective of this study. Such journal categories are not available through PubMed but available through Scimago, which is a portal that includes the journals contained in the Scopus® database (Elsevier B.V.).

In this study, all Arab countries: KSA; Egypt; Jordan; Lebanon; Qatar; Bahrain; Kuwait; Morocco; Tunisia; Syrian Arab Republic (SAR); UAE; Iraq; Sudan; Yemen; Algeria; Comoros; Djibouti; Libya; Mauritania; Oman; Somalia, except Palestine, were used as country keys followed by "WC = (Public, Environmental & Occupational Health) as a WOS category. The search strategy was like this: (CU = (Jordan) OR CU = (Iraq) OR CU = (Syria) OR CU = (Saudi) OR CU = (Kuwait) OR CU = (Egypt) OR CU = (Yemen) OR CU = (Qatar) OR CU = (Emirates) OR CU = (Bahrain) OR CU = (Oman) OR CU = (Sudan) OR CU = (Tunisia) OR CU = (Algeria) OR CU = (Lebanon) OR CU = (Libya) OR CU = (Morocco) OR CU = (Somalia) OR CU = (Djibouti) OR CU = (Comoros) OR CU = (Mauritania)) AND WC = (Public, Environmental & Occupational Health). Palestine was excluded from search keys because the Web of Science database does not recognize Palestine as an independent state yet. However, we used city keys as a search strategy to extract data pertaining to research output from Palestine and then the results of the 2 search strategies were combined and analyzed. The search keys for Palestine looked like this: (WC = (Public, Environmental & Occupational Health) AND CI = ((Nablus) OR (Jenin) OR (Ramallah) OR (Bethlehem) OR (Tulkarem) OR (Abu dis) OR (Gaza)) AND CU = (Israel)). Furthermore, to increase the accuracy of results, research was refined and limited to original research articles and review articles because they represent the original research activities, while other types of documents like editorials, conference proceedings, and others were excluded. The time frame for the result was set as 1900 – 2012. Of course, this does not mean that there will be publications from Arab countries as early as 1900. However we set this date to retrieve as many publications as possible. The 2013 and 2014 years were excluded because they are still open for new journal issues. Finally, the authors would like to state that using this methodology will not retrieve 100% of the literature published in the field of public, environmental and occupational health in Arab countries simply because there are some international and regional health journals that are not indexed in ISI web of science. For example, *Eastern Mediterranean Health Journal* is one of the regional journals in the field of public health in which many Arab researchers did publish; however, documents published in this journal were not counted because it is not indexed in ISI Web of Science.

The Web of Science database generates a count of the total number of original articles, total citations, and the value of the *h*-index (highly cited index). The *h*-index represents the number of citations received for each of the documents in descending order, for example: *h*-index of 10 means that there are 10 items that have 10 citations or more [86, 87]. Publication activity was adjusted for Arab countries, categorized by population size and gross domestic product (GDP), which was retrieved from the online databases of the World Bank [88]. An adjustment index (AI) was calculated using the following formula: AI = [total number of publications for the country/GDP per capita of the country]*1000, where the GDP per capita = GDP/population of the country [9, 89, 90]. Scientific output was evaluated based on a methodology developed and used in other bibliometric studies [91–93]. The collected data were used to generate the following information: (a) total and trends of contributions to research during all previous years up to the set date of data analysis (December 31th, 2012); (b) Arab countries research productivity and collaboration patterns; (c) journals in which Arab world researchers published; and (d) the citations received by the publications.

### Ethical approval

The Institutional Review Board (IRB) at An-Najah National University does not require submission of an IRB application for such study. The IRB considered that there is no risk for human subjects in such publications since the data are based on published literature and did not involve any interactions with human subjects.

### Statistical analysis

Data from ISI Web of Science were exported to Microsoft Office Excel® and then transferred to the Microsoft word program. The measurements of bibliometric analysis (e.g. countries, cited articles, institutions) were converted to the rank order using the standard competition ranking (SCR). We took into consideration the top 10 ranking in each item. If the measurements of bibliometric analysis have the same ranking number, then a gap is left in the following ranking numbers. The journal’s impact factors (IF) were evaluated using the Journal Citation Report (JCR; Web of Knowledge) 2012 science edition by Thomson Reuters (New York, NY, USA).

## Results

**Figure 1:**
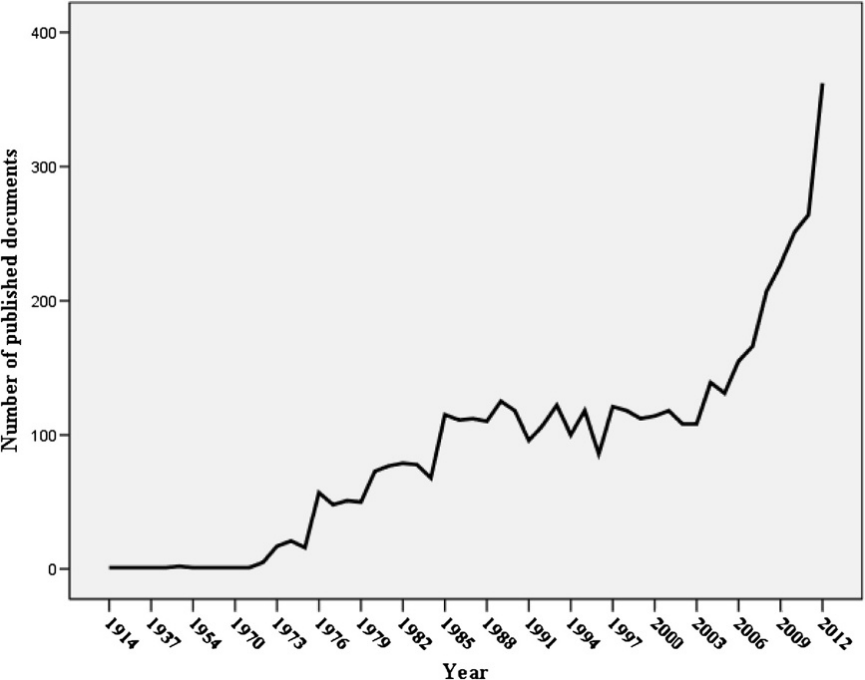
**Annual research output of public, environmental and occupational health in Arab countries.**

The total number of documents retrieved was 4673. The language used in most of the published documents was English (4433; 94.86%). The annual number of published documents indicated that research activity remained low but steadily increasing. A sharp upward increase in research activity was seen after 2002 (Figure 1). Areas of research interest of the 4673 published documents were mainly tropical medicine (1862; 39.85%) followed by environmental sciences/ecology (592; 12.67%) and infectious diseases (330; 7.06%). Table 1 shows top 10 research areas of published documents from Arab countries in the field of public, environmental and occupational health. Analysis of data based on each country’s productivity showed that Egypt (1200; 25.86%) had the highest quantity of published documents followed by KSA (738; 15.79%) and Sudan (602; 12.88%). More than half (54%) of public, environmental and occupational health research from Arab countries came from Egypt, KSA, and Sudan (Table 2). Further analysis of research activity using number of population and national income to standardize results showed that Somalia, Egypt, and Sudan had the highest productivity as measured by adjustment index (Table 2). The total number of citations of documents from Arab countries, at the time of data analysis (June 8th, 2014), was 51779 with an average citation of 11.08 per document. Of the 4673 documents considered for the *h*-index, 67 had been cited at least 67 times at the time of data analysis. Analysis showed that documents published from Egypt had the highest *h* index (51) followed by those published from Sudan (43) and KSA (32).

**Table 1:** **Research areas of the 4673 public, environmental and occupational health research documents published from Arab countries**

SCR ^a^	Research area	Number of published document N = 4673 (%)
**1** ^**st**^	Tropical Medicine	1862 (39.85)
**2** ^**nd**^	Environmental Sciences Ecology	592 (12.67)
**3** ^**rd**^	Infectious Diseases	330 (7.06)
**4** ^**th**^	Parasitology	292 (6.25)
**5** ^**th**^	Nuclear Science Technology	204 (4.37)
**5** ^**th**^	Radiology Nuclear Medicine Medical Imaging	204 (4.37)
**7** ^**th**^	Biomedical Social Sciences	148 (3.17)
**8** ^**th**^	Toxicology	141 (3.02)
**9** ^**th**^	Pathology	138 (2.95)
**10** ^**th**^	Engineering	103 (2.20)

*Abbreviations*: *SCR* Standard Competition Ranking.

^a^Equal research areas have the same ranking number, and then a gap is left in the ranking numbers.

**Table 2:** **Citation analysis, research collaboration and adjustment index (AI) for each Arab country in the field of public, environmental and occupational health**

Country	Number of documents N = 4673 (%) ^a^	Total citations	Mean citation per document	***h***index	Number of non-Arab collaborating countries	Number (%) ^b^ of documents with international authors	AI ^c^
Egypt	1200 (25.86)	15938	13.28	51	99	695 (57.92)	376.46
KSA	738 (15.79)	6233	8.45	32	72	249 (33.74)	29.36
Sudan	602 (12.88)	8983	14.92	43	84	331 (54.98)	381.05
Lebanon	363 (7.77)	4099	11.29	30	74	184 (50.69)	37.40
Tunisia	331 (7.08)	2615	7.90	24	80	133 (40.18)	78.15
Kuwait	274 (5.86)	3068	11.20	25	57	123 (44.89)	5.53
Jordan	232 (4.96)	2221	9.57	22	61	107 (46.12)	46.92
UAE	219 (4.69)	2309	10.54	23	54	127 (57.99)	5.78
Morocco	181 (3.87)	1658	9.16	21	85	106 (58.56)	61.33
Algeria	150 (3.21)	804	5.36	15	57	48 (32.00)	28.05
Iraq	131 (2.80)	1174	8.96	17	24	39 (29.77)	20.29
Syria	78 (1.67)	1418	18.18	22	41	59 (75.64)	23.72
Palestine	69 (1.48)	559	8.10	12	32	40 (57.97)	69.53
Libya	68 (1.46)	627	9.22	16	24	31 (45.59)	6.71
Oman	68 (1.46)	587	8.63	14	30	39 (57.35)	3.22
Qatar	59 (1.26)	347	5.88	11	22	36 (61.02)	0.71
Bahrain	58 (1.24)	370	6.38	12	22	27 (46.55)	2.63
Somalia	52 (1.11)	753	14.48	17	17	52 (100.00)	578.41
Yemen	58 (1.24)	424	7.57	13	36	39 (67.24)	38.80
Mauritania	21 (0.45)	196	9.33	8	13	12 (57.14)	18.98
Djibouti	9 (0.19)	91	10.11	4	8	8 (88.89)	9.13
Comoros	8 (0.17)	179	22.38	6	13	6 (75.00)	9.63

^a^Total exceeds 100% because data are overlapping due to multiple collaborations.

^b^Percentage of documents with international authors from the total number of documents for each country.

^c^An AI was calculated using the following formula: AI = [total number of publications for the country/GDP per capita of the country]*1,000. Where: GDP per capita = GDP/population of the country.

*Abbreviations*: *KSA* Kingdom of Saudi Arabia, *UAE* United Arab Emirates.

Collaboration between Arab countries and non-Arab countries in public, environmental and occupational health research was evident. All published documents (100.00%) from Somalia were made possible through collaboration with international authors. Iraq and Algeria had the lowest collaboration with 32.00% of Algerian published documents and 29.77% of Iraqi published documents were made through collaboration with non-Arab investigators. The study identified 2036 (43.57%) documents with 141 countries in Arab- non-Arab country collaboration analysis. Arab countries actively collaborated with authors from the United States of America (n = 910, the highest number recorded), followed by the England (n = 371), and France (n = 228) (Table 3). By region, Arab countries collaborated most with countries in Western Europe (22.91%) followed by North America (21.04%).

**Table 3:** Collaborations between Arab countries and foreign countries in public, environmental and occupational health*

Collaborating countries ^a^	Number of documents (%)	Collaborating countries	Number of documents	Collaborating countries	Number of documents
*Arab - North America*	983 (21.04%)	*Arab – Africa*	156 (3.33%)	*Arab - Latin America*	98 (2.09%)
USA	910 (19.47)	Kenya	57 (1.22)	Brazil	31 (0.66)
Canada	103 (2.20)	Nigeria	28 (0.60)	Peru	23 (0.49)
*Arab - Western Europe*	*1071 (22.91%)*	South Africa	19 (0.41)	Argentina	16 (0.34)
England	371 (7.94)	Uganda	19 (0.41)	Colombia	14 (0.30)
France	228 (4.88)	Ghana	22 (0.47)	Cuba	12 (0.26)
Switzerland	123 (2.63)	Tanzania	11 (0.24)	Mexico	18 (0.39)
Spain	34 (0.73)	Burkina Faso	11 (0.24)	Chile	7 (0.15)
Netherlands	95 (2.03)	Malawi	11 (0.24)	Uruguay	4 (0.09)
Germany	92 (1.95)	Cameroon	10 (0.21)	Costa Rica	3 (0.06)
Sweden	79 (1.69)	Zimbabwe	8 (0.17)	Barbados	3 (0.06)
Italy	68 (1.46)	Ethiopia	8 (0.17)	*Asiatic Region*	*279 (5.97%)*
Belgium	54 (1.16)	Senegal	9 (0.19)	Japan	41 (0.88)
Scotland	52 (1.11)	Congo	6 (0.13)	India	66 (1.41)
Denmark	47 (1.01)	Mali	6 (0.13)	Indonesia	31 (0.66)
Finland	25 (0.54)	Niger	5 (0.11)	Malaysia	26 (0.56)
Norway	23 (0.49)	Zaire	5 (0.11)	Bangladesh	20 (0.43)
Austria	16 (0.34)	Cote Ivoire	3 (0.04)	Philippines	19 (0.41)
Greece	16 (0.34)	*Arab - Pacific Region*	*71 (1.51%)*	Afghanistan	4 (0.09)
Fed Rep Ger	12 (0.26)	Australia	64 (1.37)	Pakistan	46 (0.98)
Portugal	10 (0.21)	New Zealand	9 (0.19)	Thailand	46 (0.98)
Cyprus	8 (0.17)	*Arab - Eastern Europe*	*58 (1.24%)*	Peoples R China	45 (0.96)
Ireland	8 (0.17)	Poland	10 (0.21)	South korea	17 (0.36)
North Ireland	8 (0.17)	Czech Republic	8 (0.17)	Vietnam	12 (0.26)
Wales	7 (0.15)	Russia	6 (0.13)	Singapore	10 (0.21)
Luxembourg	4 (0.09)	Hungary	5 (0.11)	Nepal	9 (0.19)
Malta	3 (0.06)	Estonia	4 (0.09)	Uzbekistan	4 (0.09)
*Arab - Middle East*	*46 (0.1%)*	Macedonia	3 (0.06)	Sri Lanka	4 (0.09)
Turkey	11 (0.24)	Yugoslavia	3 (0.06)	Cambodia	3 (0.06)
Israel	39 (0.88)			Mongol Peo Rep	3 (0.06)
Iran	7 (0.15)				

^a^The study identified 2036 (43.57%) documents with 141 countries in Arab- non-Arab country collaboration analysis.

*Each of the following countries in Africa had ≤ 2 document of collaboration with Arab researchers (Afrass Issas, Mozambique, Lesotho, Mauritius, Madagascar, Namibia, Senegambia, Angola, Zambia, Seychelles, Western Sahara, Fr. Polynesia, Albania, Armenia, Azerbaijan, Romania, USSR, Lithuania, Bosnia Herceg, Bulgaia, Slovakia, Slovenia, Ukraine, Serbia, Croatia, Czechoslvokia, Papua N Guinea, Iceland, Liberia, Gabon, Gambia, Sierra Leone, Togo, Burundi, Benin, Botswana, Guinea, Venezuela, Haiti, Nicaragua, Paraguay, Guatemala, Dominican Rep, Jamaica, Ecuador, El Salvador, Grenada, Panama, Trinid Tobago, Taiwan, Tajikistan, Kazakhstan, Kyrgyzstan, Laos, Myanmar, Hong Kong, Hong Kong).

Table 4 lists top 10 journals in which public, environmental and occupational health research documents were authored or co-authored by investigators from Arab countries. More than half of the top 10 journals were in the field of tropical medicine. One journal of the top 10 is in French language while the others were in English language. Analysis showed that a total of 481 (10.29%) documents were published in epidemiology journals, 103 (2.2%) were published in preventive medicine journals, 467 (9.99%) documents were published in environmental journals, and 105 (2.24%) were published in occupational health journals. The remaining documents (3736; 79.94%) were published in journals pertaining to public health.

**Table 4:** **List of top 10 journals in which the 4673 documents were published along with their impact factor**

SCR	***Journal***	Number of documents (%) N = 4673	IF ^a^
**1** ^**st**^	*American Journal of Tropical Medicine and Hygiene*	424 (9.07)	2.534
**2** ^**nd**^	*Transactions of the Royal Society of Tropical Medicine and Hygiene*	372 (7.96)	1.823
**3** ^**rd**^	*Annals of Tropical Medicine and Parasitology*	290 (6.21)	1.313
**4** ^**th**^	*Journal of Tropical Medicine and Hygiene*	223 (4.77)	NA
**5** ^**th**^	*Tropical and Geographical Medicine*	176 (3.77)	NA
**6** ^**th**^	*Radiation Protection Dosimetry*	143 (3.06)	0.909
**7** ^**th**^	*Bulletin de la Societe de Pathologie Exotique*	138 (2.95)	NA
**8** ^**th**^	*Journal of Environmental Science and Health Part B Pesticides Food Contaminants and Agricultural Wastes*	132 (2.83)	1.211
**9** ^**th**^	*Bulletin of the World Health Organization*	127 (2.72)	5.250
**10** ^**th**^	*Tropical Medicine International Health*	96 (2.05)	2.938

*Abbreviations*: *SCR* Standard Competition Ranking, *NA* not available, *IF* impact factor.

^a^The impact factor was reported according to Institute for Scientific Information (ISI) journal citation reports (JCR) 2012.

Finally, analysis of most research productive institutions in Arab countries showed that University of Khartoum with 343 documents (7.43%) ranked first followed by American university of Beirut and King Saud University with 292 (6.25%) and 270 (5.78%) documents respectively. The world health organization is an active partner in research in Arab countries in the field of public, environmental and occupational health. A total of 141 documents were co-authored by investigators from WHO. Furthermore, investigators from ministries of health from various Arab countries were present in 248 documents suggesting active participation of governmental bodies in public health research. Table 5 showed the top 10 productive institutions in public, environmental and occupational health in Arab countries.

**Table 5:** **Top 10 productive institutions in the field of public, environmental and occupational health research**

SCR	Institution in Arab countries	Number of documents (%) N = 4673
**1** ^**st**^	University of Khartoum	343 (7.34)
**2** ^**nd**^	American University of Beirut	292 (6.25)
**3** ^**rd**^	King Saud University	270 (5.78)
**4** ^**th**^	Kuwait University	183 (3.91)
**5** ^**th**^	University of Alexandria	166 (3.55)
**6** ^**th**^	Ain Shams University	159 (3.40)
**7** ^**th**^	Cairo University	108 (2.31)
**8** ^**th**^	United Arab Emirates University	105 (2.25)
**9** ^**th**^	National Research Center	86 (1.84)
**10** ^**th**^	King Faisal University	78 (1.67)

*Abbreviations*: *SCR* Standard Competition Ranking.

## Discussion

In this study, we analyzed a total of 4673 documents published from Arab countries in the field of public, environmental and occupational health. These documents were extracted from ISI web of science database. Despite certain points of weaknesses of ISI WoS in this regard, our study does give a clear picture and a close approximation of the quantity, quality, citation analysis, and international collaboration in research activity of documents in the field of public, environmental and occupational health in Arab countries. Furthermore, this study does establish a baseline data for future analysis and comparison in the field of public, environmental and occupational health. This becomes important given the political instability and reform going on in many Arab countries.

Our study showed that all Arab countries, including countries like Somalia, have made variable contribution to public, environmental and occupational health research. The contribution of Arab countries to the field has been noticeable and evident all through the past years with particular growth after 2000. Interest of all Arab countries in this field indicates that governments and researchers are aware of the importance of public, environmental, and occupational health problems in Arab countries and are willing to address such problems. However, some Arab countries like Egypt and KSA have made the greatest research efforts. It was not surprising that Egypt and KSA were in the top of the list in quantity of research publications in the field of public, environmental, and occupational health. Similar findings were obtained when other medical fields were investigated and analyzed using bibliometric analysis [3, 89]. Based on our findings, countries with high national income or large population size, as was observed for Egypt and KSA; have the highest quantity of research output. It was surprising to us that Sudan came on the third position regarding the quantity of research publications. Actually Sudan came second when publication quality was measured by *h*-index. Furthermore, when data were re-calculated using adjustment index, Sudan was still among the top productive countries in the field of public, environmental and occupation health in Arab countries. Our data also indicated that University of Khartoum was top productive institute in the Arab world regarding research activity in this field. Several reasons could be cited for this. First, Sudan and Egypt showed high percentage of documents with international authors. Another potential reason for Sudan and Egypt to be among top leading countries in research in this field is the high prevalence of tropical parasitological diseases in these two countries [94, 95]. A final word here is the score of Somalia in AI which was the highest among all Arab countries. This is easily explained by the finding that all publications from Somalia were with international collaboration particularly physicians visiting Somalia with non-governmental organization or through the world health organization team based in Somalia and African countries.

Researchers in Arab countries mainly collaborated with researchers from the United States of America, England and France. This may be due to the fact that most Arab academics and researchers were educated and trained in Europe and USA and few have academic or research ties with Asia, Africa, Latin America and other world regions. International collaboration is considered an advantage to Arab investigators. Actually, some Arab investigators had built research bridges with leading public health journals like Lancet and have succeeded in publishing series of articles regarding health situation in certain Arab countries [96–99]. A study showed that investigators with international collaborations produce higher quality articles compared with those who do not [43]. Furthermore, a study showed that highly cited articles are usually authored by a large number of scientists and often involving international collaboration [100]. Highly cited articles positively contribute to the *h*-index of the individual author and to the institution and country [101–104]. A study indicated that opportunities provided by *The Lancet* seem likely to encourage research and publication by individuals Arab investigators who have not traditionally had many chances to compete internationally [105].

In our study, we used citation analysis to measure the quality of publications from Arab countries. Citation is one of the key indicators of research quality and researchers need to be aware of mechanisms that might enhance citations of published articles. Therefore, Arab researchers in the field of public, environmental and occupational health should be encouraged to build bridges with other local, regional and international researchers in order to increase citations and therefore quality of their publications. This will improve research capacity and will reflect positively on the current and future health research in Arab world. The *h*-index was developed to overcome the main disadvantages of other bibliometric indicators, such as total number of papers or total number of citations. The *h*-index simultaneously measures the quality and quantity of scientific output. The *h*-index is one of the most commonly used indicators of research quality. However, *h*-index has certain disadvantages. For example, different databases can give different values of *h*-index and therefore each database has pros and cons when measuring the *h*-index [106–108]. Criticisms have also been addressed to the use of *h*-index as a marker of publication quality and citation. For example, the *h*-index does not consider the context of citations, the number of authors in the document, and gives equal values for book citation and research citation. Therefore, the *h*-index has lesser predictive accuracy and precision than mean citations per paper, although this is controversial [109, 110]. In our study, the h-index and mean citation per document were relatively high suggesting that publications from Arab countries have international audience and read and cited by other researchers. The *h*-index is commonly used to measure the performance of investigators and scientists and is based on the scientist’s most cited articles and the number of citations that they have received in other publications. However, the *h*-index can also be applied to measure the performance of a group of scientists, such as a department or university or country, or a peer reviewed journal [111–113].

Research areas of published documents in the field of public, environmental and occupational health from Arab countries was mainly in tropical medicine. Actually tropical medicine, infectious diseases and parasitological research areas constituted more than 50% of the published documents from Arab countries. Furthermore, the results of our study showed that journals in the field of tropical medicine have a good share from public, environmental and occupational health publications from Arab countries. Interest in research regarding tropical diseases in Arab countries is expected given the high prevalence of such diseases and their heavy health impact on several Arab countries [114–118].

It is noteworthy that some Arab authors have succeeded in publishing in high-quality journals like *Bulletin of the World Health Organization*. International collaborations might have helped Arab researchers to publish in journals with a high IF. Definitely, most Arab investigators are keen to publish in high impact journals. However, due to lack of funding, lack of training, lack of national research policy in most Arab countries hinder many Arab investigators to publish in high impact journals. The advantage of publishing in leading and high impact journals is attraction of large audience from international bodies and consequent funding and collaboration. Actually, some leading health journals took the initiative and encouraged many Arab researchers to publish through sponsoring annual regional conferences and subsequent publication of submitted abstracts into special issues [119].

As was expected, the documents in public health field has the greatest share of publications while those pertaining to occupational, environmental, prevention medicine and epidemiology constituted approximately 20% of the total publications. A study in developing countries indicated that occupational health research is neglected in these countries and researchers in developing countries in the field of occupational health should focus less on the workplace and more on the worker in his or her social context [120]. Actually, occupational injuries in some Arab countries have been reported to be high [62]. One potential reason for this neglect of occupational health research in Arab countries is the lack of experts or lack of awareness about the importance of this discipline [121].

Finally, it is important to mention here that several bibliometric studies about public health in general were published from non-Arab countries. One study showed that USA is leading the world in the fields of Preventive Medicine, Occupational/Environmental Medicine and Epidemiology, and the contribution of USA researchers to the field of Public Health is outstanding. On the other hand, less developed world regions are still lagging behind in the field and governments should support researchers in order to improve scientific production and advancement of knowledge in their countries [67]. A second study from India indicated that public health research output is increasing in India but the distribution of research topics and the quality of research reports continue to be unsatisfactory and that health policy makers need to address deficits in public health research in order to reduce the very large disease burden in India [69]. A third study in Africa showed regional variation in research output in the field of public health. The authors of the study indicated that this might assist policymakers to facilitate the advancement of public health research in different regions of Africa, and could be useful for international organizations in identifying needs and to allocate research funding [66].

### Limitation

This study is not without limitations. First of all, we used ISI WoS category criteria for data extraction. Articles published in journals not categorized in the public, environmental and occupational health category were not included. Furthermore, journals that were not indexed in ISI WoS were not counted in the analysis. For example, research reports published in *Eastern Mediterranean Health Journal* were not counted because this journal is not indexed in ISI WoS. Furthermore, the methodology developed in this article did not distinguish between public, environmental and occupational health.In addition, some international journals do not recognize countries like Palestine as an independent state and publications from Palestine may be affiliated with Israel as a country. Therefore, some publications from Palestine might be missed from our analysis.

## Conclusion

This study, to the best of the authors’ knowledge, is the first detailed analysis of research output in the field of public, environmental and occupational health from Arab countries. This paper’s main goals was to direct attention and open the doors for a scientific discussion among professionals and academics to direct research in this field to bridge the gap and increase visibility of health problems in Arab countries. Health policy makers in Arab countries should identify gaps in public health research and allocate funds to carry out studies to fill these gaps. It is hoped that such a study will serve those who draw future plans regarding public health to direct research into research areas mostly needed based on the changing political and social atmosphere in the Arab world. Both communicable and non-communicable disease problems need to be made visible to the international health organizations as well as governmental health bodies in Arab countries in order to set priorities for funding and financial allocations. Furthermore, this study should enable academics and investigators to search for most potential international investigators who share the same public health interest in order to cooperate successfully in research and publications. International publications should be directed toward making serious and common health problems in Arab countries more visible to internal and external audience in order to take an action by those in the authority. Definitely, topics like environmental health and preventive medicine need to be over emphasized, not only at the research level but also at the academic and governmental levels. The present data show a good start and promising rise for research activity in public health in Arab countries. However, research output in environmental and occupational health is still low. Studies in the field of preventive medicine as well as epidemiological studies in Arab countries are particularly needed. Investing in more international and national collaborative research projects should be highly encouraged. One potential method to broaden such international cooperation is through academic scholarships, exchange visits of graduate students as well as investigators. Establishing national specialized institutions for public, environmental and occupational health at the level of the entire Arab world is helpful and will make cooperation, even among Arab countries themselves, stronger and better. Such a public health institution could cooperate with the World Health Organization in increasing awareness regarding recent public health problem in the Arab world and could establish research connections with international researchers and could issue a specialized journal that will be the voice of public health specialists in Arab countries.

## Abbreviations

AI: Adjustment index
GDP: Gross domestic product
IF: Impact factor
IRB: Institutional review board
IDF: International diabetes federation
ISI: Institute for scientific information
JCR: Journal citation reports
KSA: Kingdom of Saudi Arabia
WoS: Web of science
WHO: World Health Organization
SCR: Standard competition ranking.
